# 511 Mobilization with Femoral Catheters in the Burn Intensive Care Unit: A Case Series

**DOI:** 10.1093/jbcr/irad045.108

**Published:** 2023-05-15

**Authors:** Audrey O'Neil, Cassandra Rush, David Roggy, Brett Hartman

**Affiliations:** Richard M. Fairbanks Burn Center, Indianapolis, Indiana; Eskenazi Health, Indianapolis, Indiana; RIchard M. Fairbanks Burn Center at Eskenazi Health, Indianapolis, Indiana; Indiana university/Eskenazi Health, Indianapolis, Indiana; Richard M. Fairbanks Burn Center, Indianapolis, Indiana; Eskenazi Health, Indianapolis, Indiana; RIchard M. Fairbanks Burn Center at Eskenazi Health, Indianapolis, Indiana; Indiana university/Eskenazi Health, Indianapolis, Indiana; Richard M. Fairbanks Burn Center, Indianapolis, Indiana; Eskenazi Health, Indianapolis, Indiana; RIchard M. Fairbanks Burn Center at Eskenazi Health, Indianapolis, Indiana; Indiana university/Eskenazi Health, Indianapolis, Indiana; Richard M. Fairbanks Burn Center, Indianapolis, Indiana; Eskenazi Health, Indianapolis, Indiana; RIchard M. Fairbanks Burn Center at Eskenazi Health, Indianapolis, Indiana; Indiana university/Eskenazi Health, Indianapolis, Indiana

## Abstract

**Introduction:**

Femoral catheters are a known barrier for ICU therapy sessions and mobility progression due to the anatomical location and potential risk of complications. Safety and feasibility of mobilization has been studied in ICU populations but has not been explored specifically within the burn population. Burn survivors in the ICU have challenges in terms of line access and are at high risk of complications concerning thrombosis A previous retrospective study found that 21% of mobility sessions within the burn ICU on vented patients were limited due to line limitations. This case review was completed following implementation of new mobility guidelines allowing the mobilization of patients with femoral catheters within the burn ICU.

**Methods:**

This retrospective case series was completed in the first year following implementation of new femoral catheter mobility guidelines within a 15-bed adult burn unit. Burn therapy notes were reviewed for burn admissions with at least one femoral catheter in place, including arterial and central lines. Mobility was recorded based on achieved activity levels achieved during each session. Passive interventions (stretching, ROM, and positioning) were excluded.

**Results:**

13 patients were admitted with a total of 55 femoral catheters (8 arterial, 46 central venous) treated. Patients were seen by PT and OT for a combined 417 sessions with lines in place. A total of 65 active therapy sessions occurred with 112 mobility activities including bed exercises/cycle ergometry (11), chair mode (13), cardiac chair transfer (28), tilt table (1), sitting on the side of the bed (32), standing (15), active chair transfers (8), and ambulation (4). No catheter related complications including catheter malfunctions, removal, bleeding, or thrombosis occurred during any therapy sessions.

**Conclusions:**

Active therapy sessions including out of bed activities are safe and feasible in burn ICU patients with femoral catheters. Early mobilization within ICU settings is necessary in preventing complications, combating muscle wasting, and improving long term outcomes for survivors. This study supports that the presence of femoral catheters alone should not limit the progression of mobility interventions.

**Applicability of Research to Practice:**

Using clinical judgement and specialty training, burn therapists can safely mobilize burn ICU patients with femoral catheters in place.

